# Two Cases of Human T-Lymphotropic Virus Type I-Associated Myelopathy/Tropical Spastic Paraparesis Caused by Living-Donor Renal Transplantation

**DOI:** 10.1155/2016/4203079

**Published:** 2016-09-29

**Authors:** Yasutaka Tajima, Mariko Matsumura, Hiroaki Yaguchi, Yasunori Mito

**Affiliations:** Department of Neurology, Brain Science Center, Sapporo City General Hospital, N11 W13, Chuo-ku, Sapporo 060-8604, Japan

## Abstract

In rare instances, recipients of organ transplants from human T-lymphotropic virus type I- (HTLV-I-) positive donors reportedly developed neurologic symptoms due to HTLV-I-associated myelopathy (HAM). We present herein two cases of HAM associated with renal transplantation from HTLV-I seropositive living-donors. The first patient was a 42-year-old woman with chronic renal failure for twelve years and seronegative for HTLV-I. She underwent renal transplantation with her HTLV-I seropositive mother as the donor, and she developed HAM three years after the transplantation. The second patient was a 65-year-old man who had been suffering from diabetic nephropathy. He was seronegative for HTLV-I and underwent renal transplantation one year previously, with his HTLV-I seropositive wife as the donor. He developed HAM eight months after renal transplantation. Both cases showed neurological improvements after the immunomodulating therapies. We tried to shed some light on the understanding of immunological mechanisms of transplantation-associated HAM, focusing on therapeutic strategies based on the immunopathogenesis of the condition.

## 1. Introduction

Human T-lymphotropic virus type I (HTLV-I) is a retrovirus and an etiologic agent for adult T-cell leukaemia/lymphoma (ATL) [1] and HTLV-I-associated myelopathy (HAM) [2]. Most HTLV-I infections are attributable to vertical transmission from the mother to the child or sexual transmission. Blood transfusion is also considered an efficient transmission route of the infection; accordingly, recipients of organ transplantation have been reported to develop neurologic symptoms due to HAM [3–5]. The mean latency period following cadaveric transplantation has been estimated as 3.3 years [6]. There have been fewer reported cases of HAM developing after living donor renal transplantation; latency periods ranged from 2 to 11 months [3–6]. In these cases, a more abundant viral load and the use of immunosuppressive agents have been associated with the rapid development of HAM. Here we present two cases of HAM associated with renal transplantation from HTLV-I seropositive living-donors, focusing on therapeutic strategies based on the immunopathogenesis of the condition.

## 2. Case Reports

**Figure 1:**
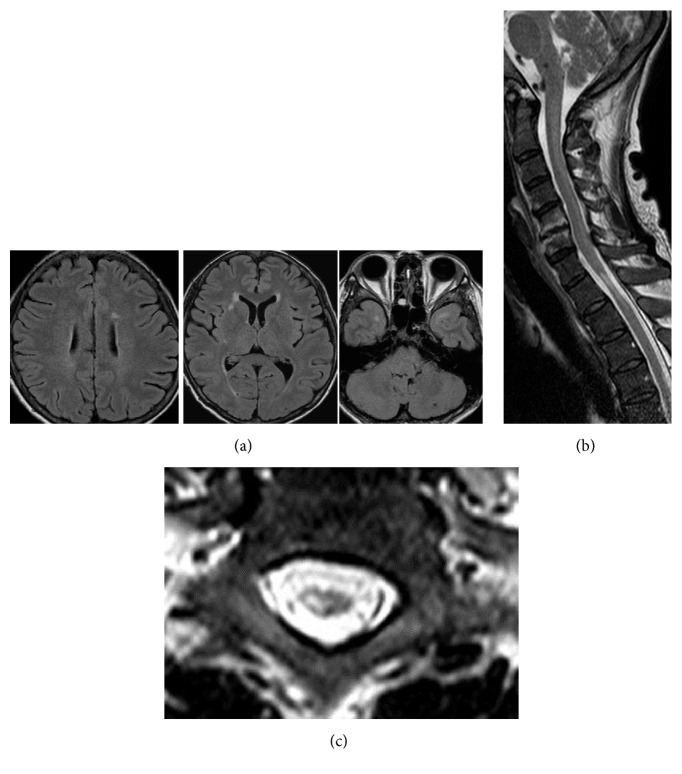
Characteristic magnetic resonance images (MRI) of Case 1. Cranial MRI showed small white matter and subcortical lesions suggestive of intracranial HTLV-I-associated myelopathy lesions (a). On spinal cord MRI examinations, a marked high signal intensity lesion was observed in the thoracic spinal cord at the level of the T1 and T2 segments (b). The spinal cord showed regional atrophy, and a T2 high-signal lesion was found mainly in the right central part of the spinal cord (c).

*Case  1*. A 42-year-old woman with chronic renal failure had been undergoing hemodialysis for 12 years and was seronegative for HTLV-I. She underwent renal transplantation 5 years previously, with her HTLV-I seropositive mother as the donor, but her renal function failed 11 months owing to toxaemia during pregnancy. Three years after the transplantation, she began experiencing difficulty with walking and attended our hospital. Neurological examinations demonstrated mild hyperreflexia in all extremities, especially the lower limbs. Spasticity of the lower limbs was evident, and the pathological reflexes were easily elicited bilaterally; she showed unstable and spastic walking. The cranial nerves and cerebellar systems were intact. She experienced mild sensory disturbance in all extremities. Her serum anti-HTLV-1 antibody titer was highly elevated (×1024); and Western blotting identified HTLV-I gP46, P19, P24, and P56 cerebrospinal fluid (CSF) antibodies. The CSF protein content was 42 mg/dL with a cell count of 14/mm^3^. Cranial magnetic resonance imaging (MRI) showed small subcortical lesions (Figure 1(a)), and MRI of the spinal cord showed focal atrophy at the level of the first and second thoracic segments (Figures 1(b) and 1(c)). In addition, a faint longitudinal T2 high signal alteration was observed in the posterior part of the spinal cord. Based on these findings, we diagnosed HAM and administered 3 million U of interferon-*α* every other day for one month. Subsequently, spasticity of the patient's lower limbs decreased. She could walk more easily, and her walking speed improved, with her Osame's Motor Disability Score (OMSD) improving from 8 to 5 after the treatment. After the treatment, repeated MRI examinations of the spinal cord were performed, and signal alterations of the atrophic spinal cord segment still remained unchanged. These observations may indicate gliosis in the spinal cord.

**Figure 2:**
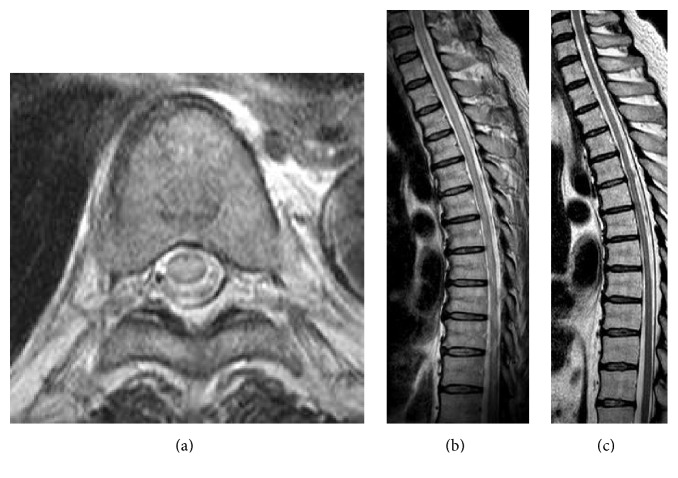
Characteristic spinal magnetic resonance images of Case 2. The thoracic spinal cord demonstrated longitudinal high-signal alterations with cord swelling prior to treatment ((a), (b)). After treatment, the spinal cord exhibited atrophic changes (c).

*Case  2*. The second patient was a 65-year-old man who had been suffering from diabetic nephropathy. He was seronegative for HTLV-I and underwent renal transplantation one year previously, with his HTLV-I seropositive wife as the donor. Eight months after renal transplantation, he presented with weakness of legs. This progressed rapidly, leaving him wheelchair-bound 12 days later. Despite receiving pulsed steroid therapy (1 g of methylprednisolone for three days), he was unable to regain his leg muscle strength, and he was therefore referred to our hospital. Neurological examinations showed flaccid paraparesis of the legs. Deep tendon reflexes were diminished, and the pathological reflexes were easily elicited bilaterally. Nerve conduction studies did not indicate any evidence of peripheral neuropathy. His serum anti-HTLV-I antibody titer was elevated (×256). The CSF protein content was 77 mg/dL with a cell count of 11 cells/mm^3^. Western blotting identified HTLV-I gP46, P19, P24, and P56 CSF antibodies. MRI of the thoracic spinal cord showed T2 high signal intensities with slight cord swelling (Figures 2(a) and 2(b)). Thus, the patient was diagnosed with HAM and was given 3 million U of interferon-*α* treatment daily for one month. As he showed little improvement, interferon-*α* therapy was restarted along with intravenous immunoglobulin (IVIg; 0.4 g/kg for 5 days). Following this, he was able to move his legs more easily and stand up with assistance. MRI examinations showed spinal cord atrophy (Figure 2(c)). The patient's OMSD improved from 10 to 8.

## 3. Discussion

There have been reports of causative correlations between transmission of HTLV-I from a carrier donor and the development of HAM in the recipient. In these cases, the use of immunosuppressants and the HTLV-I viral load have both been suggested as important for HAM development. It is thought that key roles in the pathogenic mechanisms of HAM are played by activated T cells, an exaggerated cytotoxic T lymphocyte response to HTLV-I, and induced cytokines via the activate lymphocytes; these could ultimately induce the spinal cord damage. One therapeutic strategy for HAM should therefore be to administer immunosuppressive agents. In our cases, tacrolimus and mycophenolate mofetil (MM) were used as immunosuppressants for renal transplantation. Tacrolimus binds to calcineurin and inhibits the expression of cytokine and proliferation of cytotoxic T cells. MM is an inhibitor of the type 2 isoform of inosine monophosphate dehydrogenase expressed in activated T and B lymphocytes and inhibits the proliferation of both T and B cells. Thus, both drugs could theoretically inhibit the development of HAM. However, in our cases, the use of immunosuppressants could not have significantly affected the development of HAM, given that Case 2 developed HAM within 8 months, a short latency period while taking immunosuppressive drugs. An intramuscular interferon-*α* injection was effective in both cases. In addition to this, Case 2 received pulsed steroid therapy and IVIg. Corticosteroids are known to exert beneficial effects on HAM, but IVIg has not often been tried. As IVIg has multiple mechanisms for immunoregulation, it may have an antiviral effect or ameliorate spinal cord damage in HAM. IVIg treatment in patients with HAM could be tried more frequently. It is thought that HAM develops more quickly following living-donor transplantation than after cadaveric renal transplantation, probably due to the direct transfer of a high quantity of the virus to the recipient. A high number of HTLV-I-infected cells (indicating a high quantity of the virus) may be correlated with the risk of onset, but this remains unconfirmed and further studies are required. The class I allele* HLA-A2* has been reported to have a preventive effect against development of HAM in patients infected with HTLV-I. However, the latency period for Case 2 who possessed the* A2* allele was 8 months, whereas it was 36 months for Case 1, who did not possess the allele. These results suggested that the onset of HAM after renal transplantation in our cases is not delayed by this potential immunologic defense mechanism.

In conclusion, reports of HAM onset following living-donor renal transplantation are rare, and further analysis of the pathological mechanisms and treatment strategy in this neurological disorder is needed.
